# The unrecognized potential of pet cats for studying aging and age-related diseases

**DOI:** 10.31491/apt.2021.12.069

**Published:** 2021-12-31

**Authors:** Warren Ladiges

**Affiliations:** a Department of Comparative Medicine, School of Medicine, University of Washington, Seattle, WA 98195, USA.

**Keywords:** Aging cats, age-related diseases, healthy aging, geroscience

## Abstract

Old cats develop chronic diseases similar to diseases in older people. One-fourth of American households own cats, and almost half are more than 7 years old. Cats share the same environment and are exposed to many of the same chemical stresses. In addition, genomic diversity and population stratification are similar to that occurring in people. With these comparative features, the aging cat represents a geroscience model to investigate the pathogenesis and therapeutic interventions for aging. However, cats are generally not recognized as a translational model for aging research mainly because of the lack of knowledge and appreciation within the scientific community. In addition, cat owners are not aware of any research programs designed to enhance healthy aging in their pets because none exist. Much work is needed to inform and educate the scientific community as well as cat owners about the power of aging cats as a transformative model to investigate aging and age-related diseases that will benefit both human and feline health.

The geroscience approach assumes that all diseases that affect primarily older adults have a common and major underlying cause of declining function and resilience that is part of the aging process [1]. Research is proving this concept, using a variety of clinical and preclinical approaches. For preclinical studies, animal models generally have advantages of shorter lifespans and various similarities in biological aging, and access to multiple tissues for laboratory assessments. However, current animal models of aging have one or more shortcomings such as lack of a comparable human disease, different pathogenesis of a comparable human disease, different environments than humans, lack of heterogeneity, lack of naturally occurring age-related disease, and dissimilar co-morbidity profiles compared to older people. These disadvantages provide the rationale to consider alternate animal models phylogenetically close to humans that will replicate the aging process in a relatively short period of time and provide a source of naturally occurring chronic diseases similar to humans, capable of responding to therapeutic interventions that will accurately predict positive results in people. The domestic pet cat is a novel model of aging with these attributes and deserves to be developed and validated.

The aging pet cat represents a large population available for preclinical studies. According to the 2017–2018 U.S. Pet Ownership & Demographics Sourcebook, an estimated 25 percent of American households own pet cats and the percentage older than 10 years is increasing. Feline lifespan is estimated at 15 years, although this varies based on geographic location, outdoor access, sex, breed, and other factors [2, 3]. Older cats experience chronic kidney disease, osteoarthritis, cognitive impairment, cardiovascular disease, diabetes, and cancer. The relationship of aging with these chronic disease conditions is still not well understood, but increasing age is a major risk factor. In this regard, the application of the geroscience concept for investigating diseases of aging in the cat would provide a natural model to develop scientifically sound strategies for aging intervention studies in people. Not only is the rationale for developing a naturally occurring aging model in cats based on comorbidities, but the fact that pet cats share the same environment and live in the same households as people provide an even more convincing rationale. In addition, the feline genetic architecture is the product of random mating, and as a result, genomic diversity and population stratification are similar to that occurring in people.

One striking but underappreciated and understudied condition associated with older age in pet cats is cognitive impairment (CI). While CI can be caused by a number of disease conditions, there is no evidence to suggest that cats develop neuropathology lesions very similar to neuropathology lesions associated with Alzheimer’s disease (AD) in people [4, 5]. These lesions include amyloid plaques and tau fibrillary tangles, which in combination are seen in domestic cats and humans, but generally not other mammals. The relationship of these neuropathology lesions with CI has not yet been investigated in cats. Another unique aspect of aging in cats in the development of type 2 diabetes in relation to obesity and insulin resistance similar to humans [6]. Cats respond clinically to diabetic medications including insulin just as humans do and are often on long-term treatment for this condition [7]. Hypertension is very common in older cats and, as in humans, is not well understood [8]. Chronic kidney disease is also very common in older cats [9] and complications can be a frequent cause of death or at least a driving rationale for humane euthanasia. The relationship of progressive renal failure to hypertension would be a fertile model for research on the underlying causes of hypertension. Cardiomyopathy is another common disease condition in cats [10].

In summary, cats are novel in that they develop chronic age-related disease conditions similar to the corresponding disease in older people. These diseases can be managed medically by pet cat owners for years, and thus represent similar chronic comorbidities as seen in humans. In this regard, investigating the relationship between systemic comorbidities and aging in cats will help to better clarify underlying pathophysiologic mechanisms and identify targets for aging intervention studies. So why is more research on aging not being done in pet cats? One explanation is that there is very little research funding to support pilot studies, model development, or infrastructure types of projects. This is reflected by uninformed scientific review committees that do not appreciate the high impact that a cat aging project would have. Therefore, more work is needed to inform and educate not only the scientific community but also pet cat owners who have the power in numbers to be heard. The message is that the aging pet cat fits well within the geroscience concept as a transformative model to investigate pathways of aging and effectively treat age-related diseases that would benefit both human and feline health.
